# The Effects of Transcranial Magnetic Stimulation on Tryptophan Metabolism and Depressive‐Like Behavior in Mice

**DOI:** 10.1002/brb3.71596

**Published:** 2026-07-14

**Authors:** Gao Yuan, Wang Zihao, Leng Yujia, Luan Rongrong, Zhao Xin, Yang Juanini, Liu Junchang, Feng Yuchao, Wang Peng

**Affiliations:** ^1^ Shaanxi University of Chinese Medicine Xianyang China; ^2^ Xi'an Central Hospital Xi'an China; ^3^ Johns Hopkins University in the United States Baltimore Maryland USA; ^4^ Department of Psychiatry Xijing Hospital, Air Force Medical University Xi'an China

**Keywords:** depressive‐like behavior, repetitive transcranial magnetic stimulation, tryptophan metabolism

## Abstract

**Objective:**

This research seeks to explore the effects of repetitive transcranial magnetic stimulation (rTMS) as an intervention on a depression mouse model and its impact on blood tryptophan metabolism.

**Methods:**

In this study, 24 mice were randomly divided into three groups: Sham, CUMS, and CUMS + rTMS, with each group comprising eight mice. The depression model was established using the chronic unpredictable mild stress (CUMS) method. High‐performance liquid chromatography‐tandem mass spectrometry (HPLC‐MS/MS) was employed to measure the levels of tryptophan and its metabolites in the mice's blood. The CUMS + rTMS group received rTMS treatment in addition to CUMS. Depressive‐like behaviors of mice in the CUMS group and the CUMS + rTMS group were evaluated and compared through the sucrose preference test, open field test, and tail suspension test.

**Results:**

Blood serotonin levels, a tryptophan metabolite, were notably lower in the CUMS group than in the Sham group. Conversely, the levels of 5‐hydroxyindole‐3‐acetic acid, xanthurenate, and indole‐3‐acetaldehyde were significantly higher in the CUMS group than in the Sham group. Additionally, the sucrose preference rate was lower, and the duration of immobility during the tail suspension test was longer in the CUMS group than in the Sham group; all these differences were statistically significant (*p* < 0.05). Following rTMS treatment, the CUMS+ rTMS group showed significantly lower blood levels of 5‐hydroxyindole‐3‐acetic acid, xanthurenate, and indole‐3‐acetaldehyde, while serotonin levels were significantly higher than those in the CUMS group. Compared with the CUMS group, mice in the CUMS + rTMS group showed higher sucrose preference and shorter immobility duration in the tail suspension test (*p* < 0.05).

**Conclusion:**

rTMS can affect tryptophan metabolism in the blood of mice and reduce symptoms similar to depression, providing new insights into the antidepressant properties of rTMS.

## Graphical abstract

Repetitive transcranial magnetic stimulation (rTMS) ameliorates chronic unpredictable mild stress (CUMS)‐induced depressive‐like behaviors in mice by modulating key tryptophan metabolism pathways, as evidenced by the restoration of serotonin levels and reduction of neuroactive metabolites.

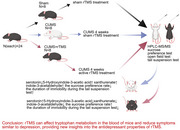

## 1 Introduction

Major depressive disorder (MDD) is the most prevalent mental health condition and ranks as the second largest contributor to the global disease burden, making it a significant public health concern (Ferrari et al. 2013). The pathogenesis of depression is highly complex, involving multiple aspects, including neuroendocrine dysfunction, neurotransmitter imbalances, and impaired neuroplasticity. Only 30% of patients with depression achieve complete remission through conventional pharmacotherapy (Rush et al. 2006). Treatment‐resistant depression (TRD) affects up to 20% of MDD patients, highlighting the increasing need for more therapeutic options (Réus et al. 2015). Repetitive Transcranial Magnetic Stimulation (rTMS) is a promising non‐invasive brain stimulation therapy that has been used to treat a variety of neurological and neuropsychiatric disorders associated with altered cortical excitability, including MDD, anxiety disorders, epilepsy, and schizophrenia (Lefaucheur et al. 2014). According to the Canadian Network for Mood and Anxiety Treatments (CANMAT) guidelines, rTMS is suggested as a primary treatment option after an unsuccessful antidepressant trial (Milev et al. 2016). Despite the effectiveness of rTMS in treating depression being demonstrated by numerous studies, the exact molecular mechanisms are still not completely known.

In recent years, research has revealed a close association between the gut microbiota and the occurrence and development of depression, with tryptophan (TRP) metabolism being one of the important pathways through which gut microbes influence the host's mood and behavior. Various products in the TRP metabolic pathway, such as indole‐3‐aldehyde (IAld) and indole‐3‐lactic acid (ILA), have been shown to be related to neuroinflammation and neuroplasticity (Zhou,Y., et al. 2023). Gut bacteria participate in the production of indole‐structured metabolites through the biodegradation of TRP. Tryptophan extracted from dietary proteins is absorbed in the small intestine and undergoes bioconversion in the colon (Lee and Lee 2010). Indole tryptophan metabolites (ITMs) generated in the gut play a role in the two‐way communication of the gut‐brain axis and are linked to neuropsychiatric disorders like attention deficit/hyperactivity disorder (ADHD) (Fernández‐López et al. 2020). Moreover, these indole metabolites impact the consumption of pleasurable foods and obesity by affecting the broader reward network, especially the amygdala‐insula and amygdala‐nucleus accumbens circuits (Osadchiy et al. 2018).

Tryptophan (TRP) metabolism primarily occurs through the serotonin pathway (5‐HTP) and the kynurenine pathway (KP). Tryptophan is converted into 5‐hydroxytryptophan in the 5‐HTP pathway, which is then changed into serotonin. Serotonin is subsequently metabolized into 5‐hydroxyindoleacetic acid. In the KP pathway, tryptophan is converted to kynurenine (Cervenka et al. 2017). Tryptophan catabolites (TRYCATs) generated through the KP influence depression by acting on brain glutamate receptors. For instance, quinolinic acid (QA), which acts as a glutamate receptor agonist, is linked to neuronal harm in brain areas that regulate mood (Forrest et al. 2015, Latif‐Hernandez et al. 2016). Conversely, kynurenic acid (KynA), which acts as an antagonist to the N‐methyl‐D‐aspartate (NMDA) receptor, engages with glutamate and is associated with neuroprotective benefits (Cervenka et al. 2017). A recent meta‐analysis found that people with MDD exhibit reduced TRP levels, with variations noted in different TRYCATs (Marx et al. 2021). Additionally, the TRP degradation pathway is implicated in numerous psychiatric disorders. For instance, patients with bipolar disorder (BD) exhibit reduced neuroprotective kynurenine metabolites in the hippocampus and amygdala (Savitz et al. 2015). The KP is crucial in treating the central nervous system and managing peripheral inflammation by decreasing TRP availability and producing oxygen radicals and strong neurotoxins (Hochstrasser et al. 2011). Some studies suggest that TRP degradation and its impact on serotonin availability may serve as potential targets for future research into alternative therapies for depression (Réus et al. 2015).

rTMS is a neuromodulatory technique that can dynamically regulate brain circuits. rTMS induces persistent changes in cortical excitability (Fitzgerald 2006). Studies have shown that, compared to sham stimulation, active rTMS is largely beneficial, producing a statistically significant reduction in depressive like symptoms such as helplessness and anhedonia (De Risio et al. 2020). In addition to its regulatory effects on cortical excitability, research has also confirmed that rTMS can modulate neurotransmitters and the gut‐brain axis. As an example, sustained rTMS affects neurotransmitters such as glutamate and GABA (Ikeda et al. 2019), and rTMS can alter the gut microbiota and medium‐ and long‐chain fatty acids in mice subjected to CUMS, thus alleviating depressive‐like behaviors (Zhou et al. 2023). It remains uncertain if rTMS can affect depressive‐like behaviors by altering TRP metabolism. The current research focused on changes in plasma TRP catabolite concentrations and assessed the impact of rTMS on TRP metabolism in mice, as well as its influence on depressive‐like behaviors.

## 2 Materials and Methods

### 2.1 Animals

Twenty‐four male C57BL/6 mice (8 weeks old, 18–22 g) were obtained from the Animal Center of Air Force Medical University (Xi'an, China). Mice were group‐housed in cages with up to five mice per cage (total 5 cages: 4 cages with 5 mice, 1 cage with 4 mice), maintained at 20–25°C under a 12 h light/dark cycle (lights on 07:00–19:00), with ad libitum access to food and water. All experimental procedures adhered to the National Institutes of Health guidelines for the care and use of laboratory animals and received approval from the Ethics Committee of the First Affiliated Hospital of Air Force Military Medical University, under project number KY20213410‐1.

### 2.2 Experimental Design

#### 2.2.1 Sample Size

Through a literature review of similar studies, it was found that in research utilizing chronic unpredictable mild stress (CUMS) to establish a mouse model of depression and assess the therapeutic efficacy of repetitive transcranial magnetic stimulation (rTMS), the sample size of each group is predominantly set within the range of 6–10 animals in the field. This sample size range reflects the conventional practices for detecting meaningful behavioral alterations in preclinical studies of stress‐induced depressive‐like phenotypes and neuromodulation interventions. In this study, the design of 8 animals per group is consistent with these established norms in the field.

## 3 Experiment

A randomization list was generated by computer (using the random number function in Microsoft Excel 2019) to allocate the 24 mice into three groups (n = 8 per group) in a 1:1:1 ratio. The experimental timeline and treatments for each group are outlined below:

Sham group (Blank Control): This group served as the untreated control. Mice were housed under standard conditions for the entire 5‐week duration without exposure to CUMS or active rTMS treatment. To control for the handling and environmental factors associated with the rTMS procedure, they received a sham rTMS treatment during the final 7 days.

CUMS group (Model Control): Mice were subjected to the chronic unpredictable mild stress (CUMS) protocol for 4 weeks to induce a depression‐like model. Following the stress period, they received sham rTMS treatment for 7 days.

CUMS + rTMS group (treatment group): Mice were subjected to the CUMS protocol for 4 weeks. This was followed by 7 days of active rTMS treatment.

Behavioral assessments for all groups were performed within 24 h after the final (seventh day) stimulation treatment. After completing the behavioral tests, blood samples were taken simultaneously from all mice via the orbital venous plexus, and serum was extracted. HPLC‐MS/MS was used to measure the levels of tryptophan and its metabolites in the serum, including l‐kynurenine, 5‐hydroxyindole‐3‐acetic acid, picolinic acid, tryptophan, xanthurenate, n‐formyl‐kynurenine, cinnavalininate, kynurenate, serotonin, indoleacetate, indole‐3‐acetaldehyde, 3‐hydroxyanthranilic acid, 3‐indoxyl sulfate, quinolinic acid, 3‐hydroxyl‐L‐kynurenine, indole‐3‐carboxaldehyde, indole‐3‐lactic acid, indole‐3‐propionic acid, and indoxyl‐β‐D‐glucuronide.

### 3.1 CUMS

CUMS (Ma et al. 2021) outlines a procedure in which mice are exposed to a variety of repeated and unpredictable stressors over a span of four weeks. These stressors include: (a) 2 h of restraint stress; (b) exposure to light overnight; (c) deprivation of food and water for 24 h; (d) a reversed light/dark cycle; (e) spending 24 h in a dirty or empty cage; (f) 2 h of interaction with another mouse; (g) 10 min of cold stress at 4°C; (h) 24 h with the cage tilted at 30°; (i) 5 min of swimming in cold water; and (j) foot shock with an intensity of 1.5 mA for 2 s, with a 1 s interval. No stressor was applied in succession. The Sham group, on the other hand, was maintained in a normal environment with unrestricted access to food and water and was not subjected to any stress.

### 3.2 rTMS Treatment

A Y064 animal stimulation coil from RuiDe, Wuhan, China, was employed, with the intensity adjusted to 40% of the coil's maximum output, approximately 1.26 T (Zhou, Cai et al. 2021). Each daily session of stimulation comprised 50 trains, each containing 20 pulses, delivered at a frequency of 15 Hz, with a 3‐second pause between trains. The coil's center was aligned above the scalp surface. For actual rTMS, the coil was aligned parallel to the mouse's parietal bone, whereas for sham rTMS, it was placed 10 cm above the head (Xue et al. 2020). Each animal was given time to get used to the background noise of the rTMS device and experienced the same duration of sham stimulation daily for a week.

### 3.3 Behavioral Tests

All behavioral assessments were performed during the light phase, specifically between 10:00 a.m. and 5:00 p.m., with mice acclimating to the lab setting for a minimum of one hour before each test. The sucrose preference test (SPT) was conducted before the open field test (OFT), and the tail suspension test (TST) was carried out 24 h after the OFT. Equipment was cleaned with 75% ethanol between tests. SPT (Liu et al. 2018): Mice were individually placed in SPT cages and given unrestricted access to a 1% sucrose (wt/vol) solution for 24 h. Following this, both water and 1% sucrose were offered simultaneously, allowing mice to drink freely for another 24 h. Mice were then deprived of water and food for 24 h before the test. During the test phase, water and 1% sucrose were placed in pre‐weighed bottles, replaced every hour, and mice were allowed to drink freely for 2 h, after which sucrose preference was calculated. OFT (Yang et al. 2016): The OFT was conducted in an open‐field arena measuring 50 cm × 50 cm × 50 cm. Mice were placed in the center of the open‐field box, and their activity was recorded and analyzed for 5 min using Top Scan (Clever Sys Inc., USA). TST (Eagle et al. 2020): Mice were suspended by their tails from a horizontal bar, 50 cm above the floor, for 6 min. Activity during the last 5 min was analyzed, with immobility defined as the absence of skeletal movement for at least 1 s.

### 3.4 Statistical Analysis

All data are expressed as mean (SD). Statistical analyses were conducted using GraphPad Prism software (version 10.1.2). Normality of data distribution for each group was assessed using the Shapiro‐Wilk test, and all datasets met the assumption of normality at α = 0.05.

The study included three experimental groups: Sham, CUMS, and CUMS + rTMS. For each key outcome measure, intergroup differences were evaluated using one‐way ANOVA. For outcomes with significant ANOVA results (*p* < 0.05), post hoc pairwise comparisons were performed using Tukey's honest significant difference (HSD) test, with adjusted *p*‐values reported to account for multiple testing.

In accordance with our predefined analytical plan, the primary focus was on two biologically meaningful comparisons: (1) CUMS vs. Sham (to validate depressive‐like phenotype induction) and (2) CUMS vs. CUMS + rTMS (to assess rTMS efficacy). Other pairwise comparisons (e.g., Sham vs. CUMS + rTMS) were not prespecified as primary outcomes and thus not emphasized.

Additionally, Pearson correlation analysis was employed to examine linear associations between plasma tryptophan metabolite concentrations and core behavioral measures (immobility time in the tail suspension test, and sucrose preference rate). All tests were two‐sided, and a *p*‐value < 0.05 was considered statistically significant.

## 4 Results

All data were complete and available for analysis, with no missing values across the experimental groups (n = 8 per group).

### 4.1 Changes in Depressive‐Like Behavior

**FIGURE 1:**
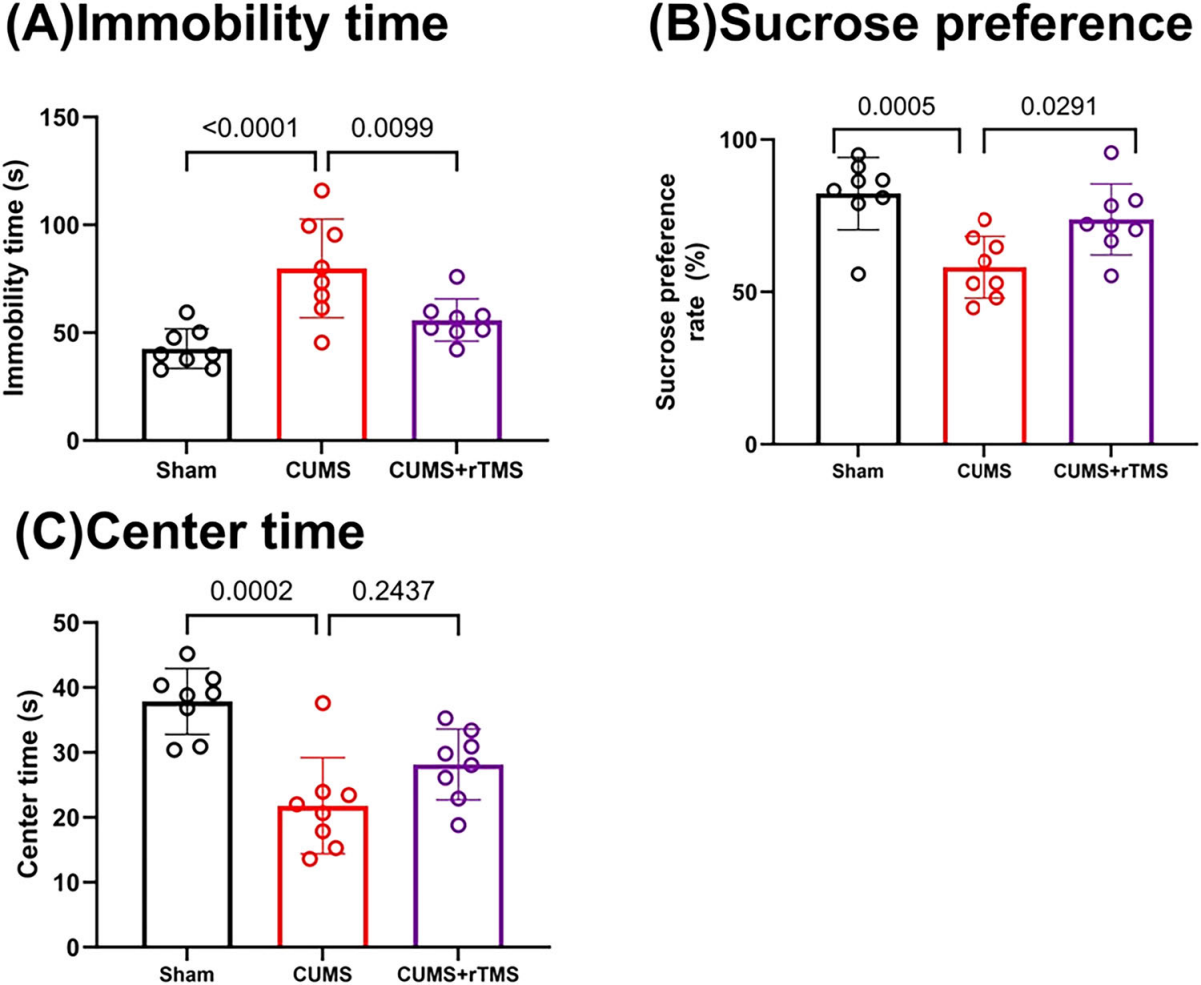
rTMS improved depressive‐like behaviors induced by CUMS. (A) Immobility time in the TST for each group. (B) Sucrose preference rate for each group of mice. (C) Time spent in the center of the OFT for each group of mice.

Compared to the Sham group, mice in the CUMS group showed significantly lower sucrose preference during the sucrose preference test (SPT) and significantly longer immobility time in the tail suspension test (TST) (CUMS vs. Sham: *p = 0.0005* for SPT; *p* < 0.0001 for TST; Figure 1A, B). In the CUMS + rTMS group compared with the CUMS group, sucrose preference was significantly higher and immobility time was significantly shorter (CUMS vs. CUMS + rTMS; *p* = 0.0291 for SPT; *p* = 0.0099 for TST; Figure 1A, B).

For the open field test (OFT), one‐way ANOVA revealed no statistically significant overall difference in total distance traveled among the three groups (*p* = 0.0832). Regarding the time spent in the center zone, a significant difference was observed among groups (*p* = 0.0003). Post hoc pairwise comparisons showed that the CUMS group spent significantly less time in the center than the Sham group (*p* = 0.0002; Figure 1C), while no significant difference was found between the CUMS and CUMS + rTMS groups (*p* = 0.2437; Figure 1C).

### 4.2 Changes in Blood Tryptophan Metabolites

A panel of 19 tryptophan metabolites was quantitatively analyzed in the blood serum. Following statistical analysis, 15 of these metabolites demonstrated significant alterations in response to the experimental interventions and are presented in Figure 2​ (subfigures A–O, where A–I represent the first panel and J–O the second panel). These 15 metabolites include l‐kynurenine, 5‐hydroxyindole‐3‐acetic acid, picolinic acid, xanthurenate, n‐formyl‐kynurenine, cinnavalininate, kynurenate, serotonin, indoleacetate, indole‐3‐acetaldehyde, 3‐indoxyl sulfate, indole‐3‐carboxaldehyde, indole‐3‐lactic acid, indole‐3‐propionic acid, and indoxyl‐β‐D‐glucuronide. The remaining four metabolites (tryptophan, 3‐hydroxyanthranilic acid, quinolinic acid, and 3‐hydroxyl‐L‐kynurenine) did not show statistically significant differences among the groups and are therefore not depicted in the figure.

FIGURE 2(A) cinnavalininate, (B) l‐kynurenine, (C) n‐formyl‐kynurenine, (D) serotonin, (E) 5‐hydroxyindole‐3‐acetic acid, (F) kynurenate, (G) picolinic acid, (H) xanthurenate, (I) 3‐Indoxyl sulfate, (J) indole‐3‐acetaldehyde, (K) indole‐3‐carboxaldehyde, (L) indole‐3‐lactic acid, (M) indole‐3‐propionic acid, (N) indoleacetate, (O) indoxyl‐β‐D‐glucuronide. Pairwise comparisons were adjusted using Tukey's HSD test, focusing on CUMS vs. Sham and CUMS vs. CUMS + rTMS.

**Figure:**
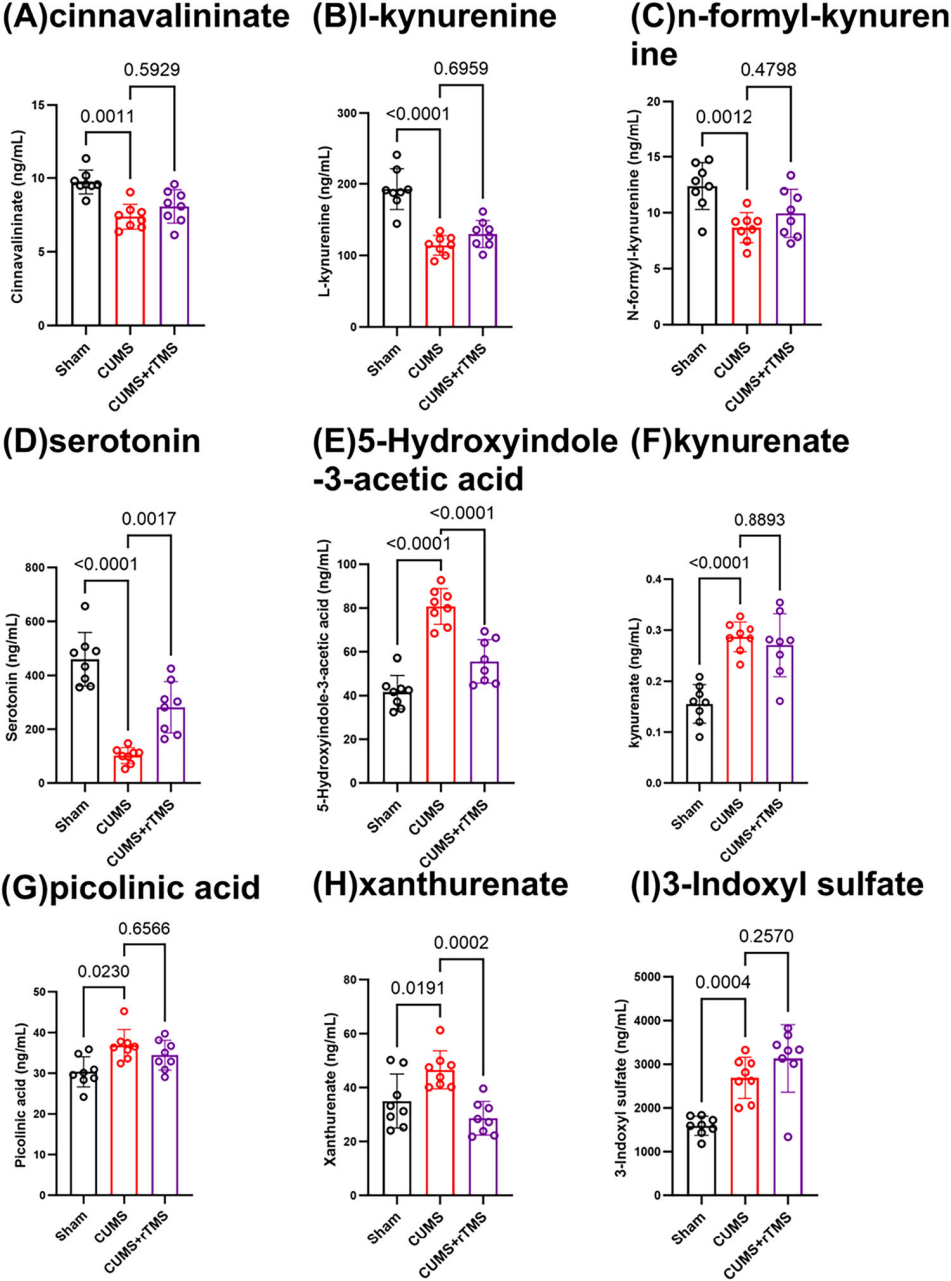

**Figure:**
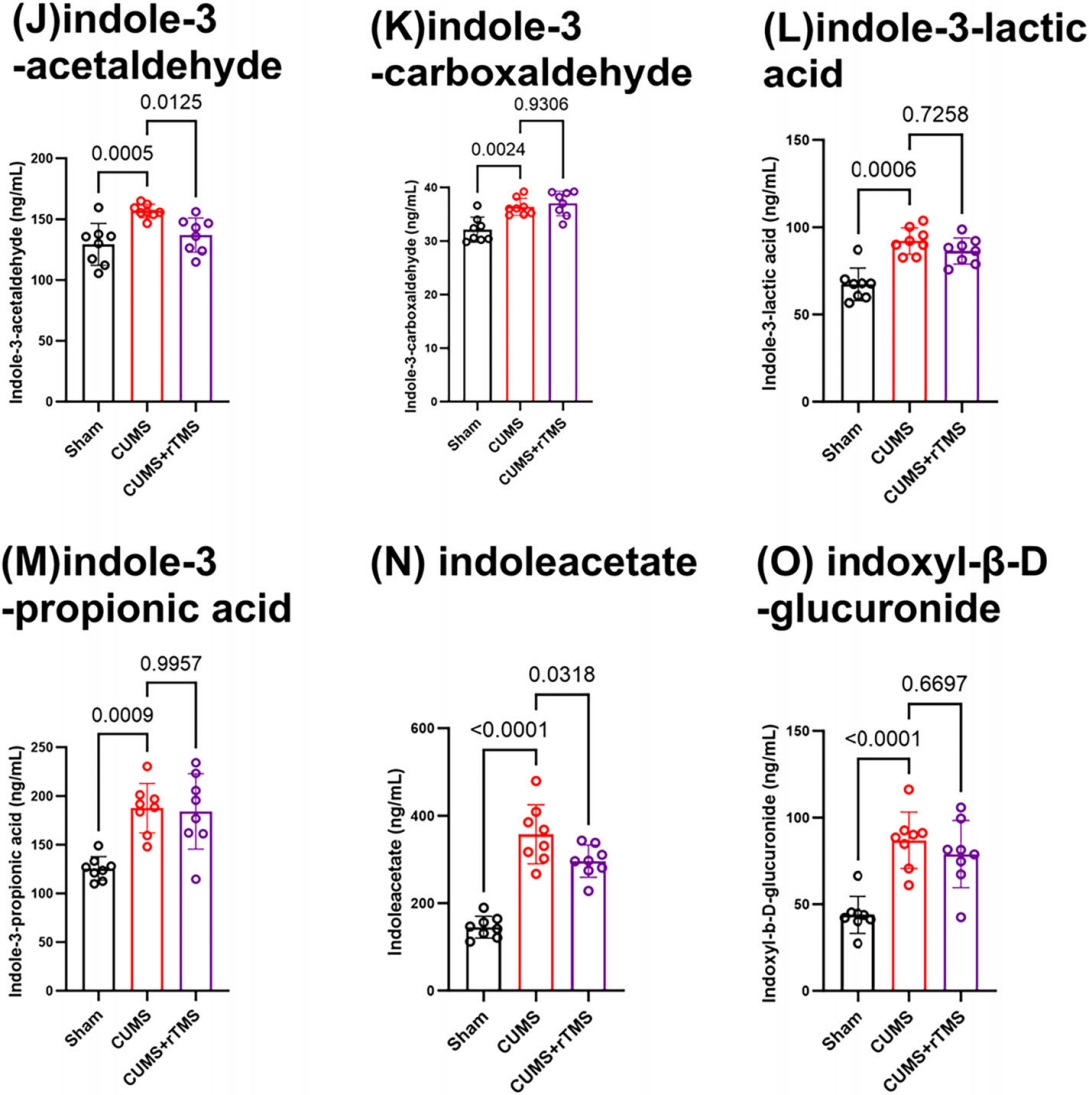

One‐way ANOVA revealed statistically significant overall differences among the three groups for all 15 tryptophan metabolites (all *p* < 0.05; specific p‐values for each metabolite are provided in Figure 2 legends).

#### 4.2.1 Comparison Of CUMS vs. Sham Groups

As illustrated in Figure 2 (subfigures A–I), the levels of cinnavalininate (A: *p* = 0.0011), l‐kynurenine (B: *p* < 0.0001), n‐formyl‐kynurenine (C: *p* = 0.0012), and serotonin (D: *p* < 0.0001) were significantly lower in the CUMS group than in the Sham group. Conversely, the levels of 5‐Hydroxyindole‐3‐acetic acid (E: *p* < 0.0001), kynurenate (F: *p* < 0.0001), picolinic acid (G: *p* = 0.0230), xanthurenate (H: *p* = 0.0191), and 3‐Indoxyl sulfate (I: *p* = 0.0004) were significantly higher in the CUMS group.

As shown in Figure 2 (subfigures J–O), additional indole‐derived metabolites were altered: indole‐3‐acetaldehyde (J: CUMS vs. Sham, *p* = 0.0005), indole‐3‐carboxaldehyde (K: CUMS vs. Sham, *p* = 0.0024), indole‐3‐lactic acid (L: CUMS vs. Sham, *p* = 0.0006), indole‐3‐propionic acid (M: CUMS vs. Sham, *p* = 0.0009), indoleacetate (N: CUMS vs. Sham, *p* < 0.0001), and indoxyl‐β‐D‐glucuronide (O: CUMS vs. Sham, *p* < 0.0001) were all significantly elevated in the CUMS group compared with the Sham group.

#### 4.2.2 Comparison of CUMS + rTMS vs. CUMS Groups

In the CUMS+rTMS group compared with the CUMS group, serotonin levels were significantly higher (Figure 2D; *p* = 0.0017), while 5‐hydroxyindole‐3‐acetic acid (E:*p* < 0.0001), xanthurenate (H:*p* = 0.0002), and indole‐3‐acetaldehyde (J:*p* = 0.0125) were significantly lower. Indoleacetate (N:*p* = 0.0318) was also lower in the CUMS + rTMS group. No significant differences were observed in other metabolites (e.g., indole‐3‐carboxaldehyde (K:*p* = 0.9306), indole‐3‐lactic acid (L:*p* = 0.7258*)*, indole‐3‐propionic acid (M:p = 0.9957), and indoxyl‐β‐D‐glucuronide (O:p = 0.6697) between these groups.

### 4.3 Correlation Between Depressive‐Like Behaviors and Blood Tryptophan Metabolite Levels

**FIGURE 3:**
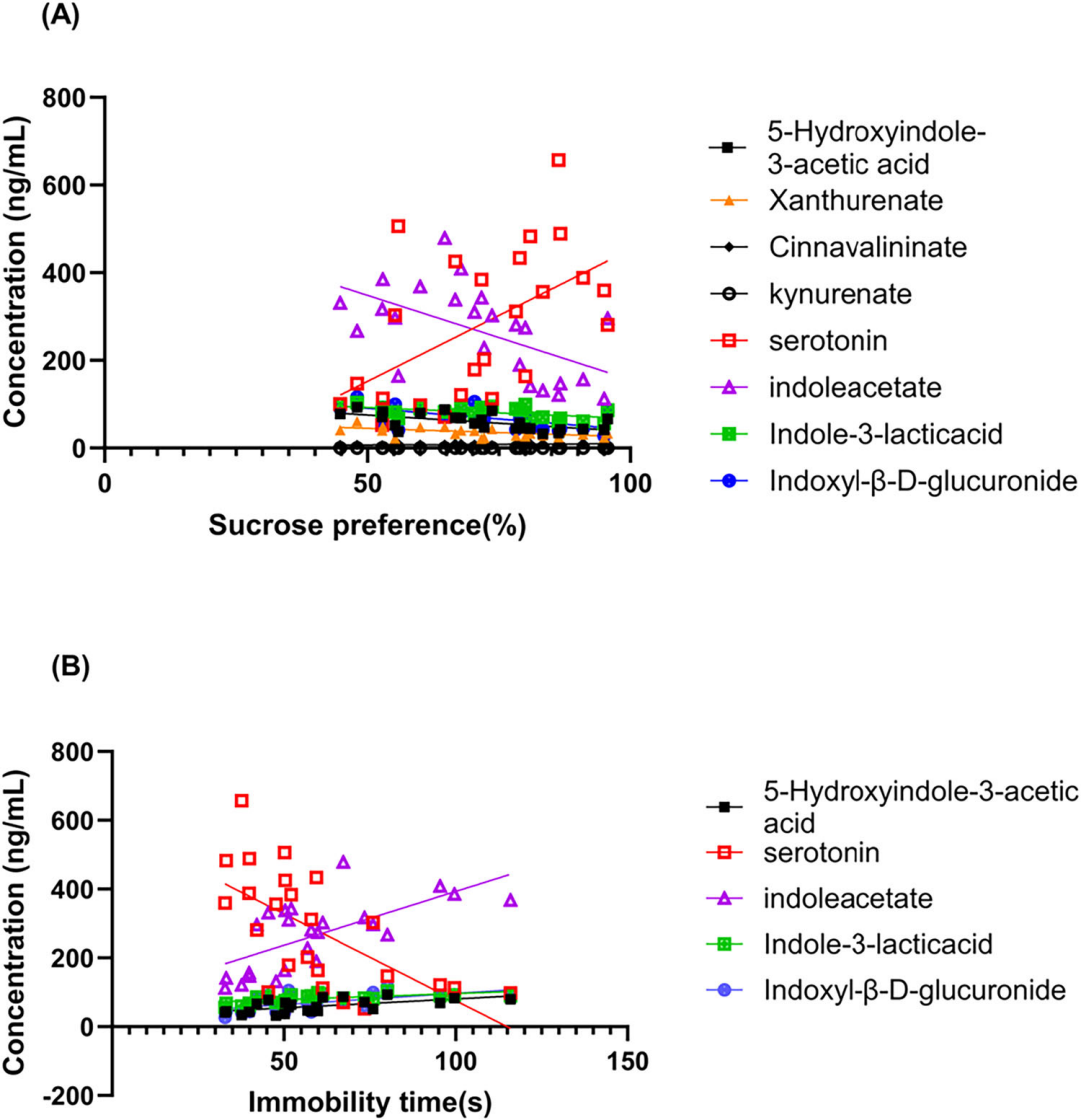
Correlation between blood tryptophan metabolites and depressive‐like behaviors. (A) Sucrose preference (%) versus metabolite concentration (ng/mL). (B) Tail suspension test (TST) immobility time (s) versus metabolite concentration (ng/mL).

Analyses were restricted to the 15 metabolites that exhibited significant group differences in Section 3.2 (Figure 2) and included all 24 mice. Results are presented for statistically significant correlations only (Figure 3).

#### 4.3.1 Correlation With Sucrose Preference (Anhedonia, SPT)

Figure 3A illustrates the linear associations between blood tryptophan metabolite concentrations and sucrose preference percentage (a behavioral indicator of anhedonia, where lower preference reflects more severe reward deficiency). Among the 15 metabolites analyzed, only serotonin and indoleacetate exhibited statistically significant linear correlations with sucrose preference:

Serotonin (red square): Showed a significant positive correlation with sucrose preference (Pearson's r = +0.56, *p* = 0.008; Figure 3A), indicating that higher serotonin concentrations were associated with increased sucrose preference.

Indoleacetate (purple triangle): Showed a significant negative correlation with sucrose preference (r = −0.55, *p* = 0.005; Figure 3A).

#### 4.3.2 Correlation With TST Immobility Time (Behavioral Despair)

Figure 3B depicts the linear associations between blood tryptophan metabolite concentrations and immobility time in the tail suspension test (a behavioral indicator of behavioral despair, where longer immobility reflects more intense hopelessness). Again, only serotonin and indoleacetate showed significant correlations:

Serotonin (red square): Exhibited a significant negative correlation with immobility time (r = −0.56, *p* = 0.004; Figure 3B).

Indoleacetate (purple triangle): showed a significant positive correlation with immobility time (r = −0.55, *p* = 0.005; Figure 3B).

No other metabolites displayed significant linear associations with immobility time in this analysis.

## 5 Discussion

In this study, we performed an extensive evaluation to assess how rTMS treatment affects blood tryptophan metabolite levels in a mouse model of depression triggered by CUMS. The CUMS procedure led to depressive‐like behaviors in mice, evidenced by a reduction in sucrose preference and an increase in immobility time during the tail suspension test. Concurrent with the behavioral changes, blood serotonin (5‐HT) levels were significantly decreased in the CUMS group, whereas 5‐hydroxyindole‐3‐acetic acid (5‐HIAA), a major serotonin metabolite, was notably elevated. Increases were also observed in xanthurenate, indole‐3‐acetaldehyde, and 3‐Indoxyl sulfate. Treatment with rTMS mitigated the depressive‐like behaviors in mice and partially reversed the CUMS induced alterations in blood tryptophan metabolite levels. These results suggest that blood TRP metabolism might play a role in the development of MDD, and the modulation of blood tryptophan metabolites by rTMS could impact the mechanisms of antidepressant action.

Recent experimental studies have shown that the production of tryptophan metabolites, including indole‐3‐acetic acid, indole‐3‐propionic acid, tryptamine, and indoxyl sulfate (IS), is linked to gut microbiota (Beloborodova et al. 2020). The relationship between the brain, gut, and microbiota is crucial for human health and disease. The gut microbiota transforms the host's diet into various metabolites, such as short‐chain fatty acids, trimethylamine N‐oxide, IS, and secondary bile acids, which affect the host's physiological functions by triggering multiple signaling pathways (Jin et al. 2019). The brain has the ability to control the gastrointestinal tract and the enteric nervous system by utilizing the parasympathetic and sympathetic divisions of the autonomic nervous system, along with the hypothalamic‐pituitary‐adrenal (HPA) axis (Mayer et al. 2015). It can also indirectly affect blood TRP metabolism by altering the gut microbiota. Therefore, neuromodulatory techniques can influence the levels of blood tryptophan metabolites.

Our results revealed changes in 5‐HT levels. CUMS induced a decrease in 5‐HT levels, which was normalized by rTMS. The serotonergic signaling system is involved in the regulation of many important bodily functions and serves as a target for antidepressant, anxiolytic, nootropic, and antimigraine medications. As an important neurotransmitter, reduced levels of 5‐HT are closely associated with depressive mood. Moreover, 5‐HT serves as a modulator of smooth muscle tone, with its effects being contingent upon its interactions with different types of central and peripheral 5‐HT receptors. Research indicates that there may be a connection between reduced platelet 5‐HT levels and depressive symptoms in individuals with schizophrenia (Peitl et al. 2016). Patients with schizophrenia who experience depressive symptoms have lower platelet 5‐HT concentrations compared to those without such symptoms, and a greater severity of these symptoms correlates with reduced platelet 5‐HT levels. This suggests that 5‐HT serum levels are involved in the mechanisms underlying depressive symptoms. According to our findings, rTMS regulated the changes in blood 5‐HT levels. This is different from previous findings (Kanno et al. 2003). A study involving rTMS to activate the frontal cortex in rats demonstrated that applying rTMS at 110% of the motor threshold nullified the rise in 5‐HT levels induced by sham stimulation. Consequently, it was inferred that acute rTMS treatment could have therapeutic potential for mood disorders. Given that the stimulation frequency, duration, and 5‐HT detection methods in our study were different from those in the previous study, the regulation of extracellular 5‐HT in the prefrontal cortex (PFC) and blood 5‐HT concentration by rTMS may differ. In summary, rTMS may alleviate depressive‐like behaviors by modulating 5‐HT levels.

Our results also observed changes in the levels of other blood tryptophan metabolites, such as increases in 5‐HIAA, xanthurenate, and indole‐3‐acetaldehyde induced by CUMS, which were improved after rTMS treatment. The findings on 5‐HIAA are consistent with previous studies (Peng et al. 2018). By applying various parameters (1/5/10 Hz, 0.84/1.26 T) of rTMS to the PFC of rats experiencing chronic unpredictable stress, it was observed that rTMS therapy elevated the levels of 5‐HT, dopamine (DA), and norepinephrine (NE), while reducing 5‐HIAA levels and alleviating depressive‐like symptoms. This suggests that 5‐HIAA, a metabolite of 5‐HT, plays a role in the antidepressant effects of rTMS. Additionally, 5‐HIAA is implicated in cognitive and endocrine functions. Consistent with this, post‐mortem studies of Alzheimer's disease (SDAT) have consistently demonstrated that hippocampal levels of 5‐HT and 5‐HIAA are markedly lower compared to controls (Baker and Reynolds 1989; Afarideh et al. 2015). Therefore, 5‐HIAA may also be involved in the mechanisms underlying cognitive impairment and inflammation, thereby indirectly affecting changes in mood and behavior.

Interestingly, while a meta‐analysis reported lower peripheral xanthurenate levels in patients with bipolar disorder (BD) (Bartoli et al. 2021), our MDD model exhibited elevated levels. This divergence suggests that xanthurenate may play distinct roles in the pathological processes of MDD versus BD. Additionally, another study identified a positive correlation between the risk of type 2 diabetes (T2D) and four metabolites in the KP—kynurenine, kynurenic acid, xanthurenate and quinolinic acid—as well as indolelactate (Qi et al. 2022). This suggests that xanthurenate may also influence mood changes by participating in endocrine functions.

Although research on the association between indole‐3‐acetaldehyde and neuropsychiatric disorders is limited, the role of indole‐3‐carboxaldehyde (I3C) in modulating depressive‐like behaviors has been demonstrated. I3C, derived from the gut microbiota, can improve depressive‐like behaviors through the gut‐brain axis pathway while regulating stress vulnerability (Chen et al. 2025). Chronic stress is a significant trigger for MDD, and therefore, I3C might play a role in antidepressant processes by influencing chronic stress. Therefore, the involvement of Indole‐3‐acetaldehyde in the pathogenesis of depression warrants further investigation.

Although the number of tryptophan metabolites whose levels were significantly regulated by rTMS in this study was limited, CUMS successfully induced changes in the levels of many tryptophan metabolites. For instance, the findings related to kynurenine align with earlier research. Patients experiencing moderate depression exhibit lower levels of kynurenine and TRP compared to healthy individuals (Kuwano et al. 2018). Therefore, TRP metabolism plays an important role in the pathogenesis of depression.

The tryptophan metabolites altered by CUMS are not only involved in the pathogenesis of depression but also implicated in other neuropsychiatric disorders, such as cognitive impairment. Uremic encephalopathy, for instance, is caused by the buildup of uremic toxins in the brain. Indole‐3‐acetate (IAA) and hippuric acid (HA) are two distinct protein‐bound uremic toxins that originate from amino acid derivatives and affect cognitive abilities. Additionally, serum IAA is linked to cognitive decline. Research indicates that individuals receiving hemodialysis (HD) are at a greater risk of experiencing cognitive impairment and dementia compared to the general population (Lin et al. 2019). Furthermore, IS, known as a uremic endothelial toxin, has been demonstrated to cause a range of negative effects in various animal models and cell cultures. In vitro studies reveal that IS triggers the activation of the endothelial aromatic hydrocarbon receptor (AhR) and pro‐inflammatory transcription factors like NF‐B or AP‐1. Additionally, IS contributes to pro‐oxidant effects by diminishing the bioavailability of nitric oxide (NO) (Lano et al. 2020). When the renal function is impaired, the accumulation of IS is toxic. Clinical studies have demonstrated that elevated levels of IS are associated with cognitive impairment and cardiovascular and renal diseases (Leong and Sirich 2016). In conclusion, IAA and IS play a role in the development of cognitive impairment, and we hypothesize that they might indirectly influence mood alterations by being involved in cognitive processes.

Moreover, indole‐3‐propionic acid (IPA), which is synthesized by gut microbiota, serves as a powerful antioxidant with properties that protect the nervous system (Karbownik et al. 2001). Since dysfunction of the gut barrier is linked to heightened activation of the mucosal immune system, IPA levels can affect the immune response of the host (Dodd et al. 2017). Existing studies have shown that indoles have different effects on gut bacteria and the host organism. Indoles may affect bacterial antibiotic resistance and biofilm formation (Lee and Lee 2010, Kim and Park 2015). Indoles modulate immune responses by boosting the inhibitory effects of the anti‐inflammatory cytokine IL‐10 and decreasing the levels of pro‐inflammatory cytokines IL‐8 and TNF. They also strengthen the gut‐blood barrier (GBB) by increasing actin cytoskeleton formation and promoting the synthesis of proteins closely related to the integrity of the intestinal epithelium (Bansal et al. 2010). Therefore, IPA may participate in neuroimmunity through antioxidant and anti‐inflammatory mechanisms, thereby indirectly influencing changes in mood and cognitive function.

In summary, rTMS, as a neuromodulatory technique, may influence TRP metabolism through multiple mechanisms, thereby improving depressive‐like behaviors. On the one hand, rTMS may modulate the release and reuptake of neurotransmitters, affecting the activity of tryptophan‐metabolizing enzymes and thus altering the levels of tryptophan metabolites in the blood. On the other hand, rTMS may indirectly influence tryptophan metabolism by regulating the neuroimmune and neuroendocrine systems. Additionally, the absence of statistically significant differences between the results of the CUMS group and the CUMS+ rTMS group may be due to insufficient sample size. Additional large‐scale and forward‐looking studies are required to validate our results.

## 6 Conclusion

The findings of this research suggest that rTMS can influence tryptophan metabolism in mice's blood and alleviate behaviors resembling depression, offering fresh perspectives on the antidepressant effects of rTMS. However, this study was conducted only in animal models, and the mechanisms and therapeutic effects of rTMS in humans require further investigation. Future research should further explore the specific regulatory mechanisms of rTMS on tryptophan metabolism.

## Author Contributions

**Wang Peng**: conceptualization, methodology, supervision, funding acquisition, project administration, resources. **Gao Yuan**: software, data curation, formal analysis, writing – original draft, investigation, validation, visualization. **Wang Zihao**: investigation, validation, writing – review and editing. **Leng Yujia**: investigation. **Luan Rongrong**: investigation. **Zhao Xin**: investigation. **Yang Juanini**: investigation. **Liu Junchang**: validation, visualization. **Feng Yuchao**: validation, visualization.

## Funding

This work was supported by the Xi’an Municipal Health Commission General Cultivation Project (2023ms03).

## Conflicts of Interest

The authors declare no conflicts of interest.

## Data Availability

The data that support the findings of this study are available from the corresponding author upon reasonable request.
